# Driving status, travel modes and accelerometer-assessed physical activity in younger, middle-aged and older adults: a prospective study of 90 810 UK Biobank participants

**DOI:** 10.1093/ije/dyz065

**Published:** 2019-04-19

**Authors:** Samantha Hajna, Tom White, Jenna Panter, Søren Brage, Katrien Wijndaele, James Woodcock, David Ogilvie, Fumiaki Imamura, Simon J Griffin

**Affiliations:** 1 MRC Epidemiology Unit, Institute of Metabolic Science, University of Cambridge, School of Clinical Medicine, Cambridge Biomedical Campus, Cambridge, UK; 2 UKCRC Centre for Diet and Activity Research (CEDAR), University of Cambridge, School of Clinical Medicine, Cambridge Biomedical Campus, Cambridge, UK; 3 Primary Care Unit, Institute of Public Health, University of Cambridge School of Clinical Medicine, Cambridge Biomedical Campus, Cambridge, UK

**Keywords:** Driving, travel modes, older adults, physical activity, UK Biobank

## Abstract

**Background:**

Associations between driving and physical-activity (PA) intensities are unclear, particularly among older adults. We estimated prospective associations of travel modes with total PA, sedentary time (ST), light-intensity PA (LPA), and moderate-to-vigorous intensity PA (MVPA) among adults aged 39–70 years.

**Methods:**

We studied 90 810 UK Biobank participants (56.1 ± 7.8 years). Driving status, specific travel modes (non-work travel; commuting to/from work) and covariates were assessed by questionnaire (2006–10). PA was assessed over 7 days by wrist-worn accelerometers (2013–15). We estimated associations using overall and age-stratified multivariable linear-regression models.

**Results:**

Drivers accumulated 1.4% more total PA (95% confidence interval: 0.9, 1.9), 11.2 min/day less ST (–12.9, –9.5), 12.2 min/day more LPA (11.0, 13.3) and 0.9 min/day less MVPA (–1.6, –0.2) than non-drivers. Compared with car/motor-vehicle users, cyclists and walkers had the most optimal activity profiles followed by mixed-mode users (e.g. for non-work travel, cyclists: 10.7% more total PA, 9.0, 12.4; 20.5 min/day less ST, –26.0, –15.0; 14.5 min/day more MVPA, 12.0, 17.2; walkers: 4.2% more total PA, 3.5, 5.0; 7.5 min/day less ST –10.2, –4.9; 10.1 min/day more MVPA, 8.9, 11.3; mixed-mode users: 2.3% more total PA, 1.9, 2.7; 3.4 min/day less ST –4.8, –2.1; 4.9 min/day more MVPA, 4.3, 5.5). Some associations varied by age (*p* interaction < 0.05), but these differences appeared small.

**Conclusions:**

Assessing specific travel modes rather than driving status alone may better capture variations in activity. Walking, cycling and, to a lesser degree, mixed-mode use are associated with more optimal activity profiles in adults of all ages.

**Key Messages**

- In previous studies, driving has been linked to lower levels of PA in young and middle-aged adults, but there is evidence that driving may facilitate PA in older adults.
- Studies investigating whether associations between travel modes and PA vary by age and that utilize objective measures of PA, across the intensity spectrum, are needed to inform intervention development and evaluation.
- Our findings suggest that walkers, cyclists and mixed-mode users of all ages are less sedentary and more physically active than car/motor-vehicle users and that assessing driving status (yes/no) alone may mask important variations in PA.

## Background

Inactivity is a major threat to health in people of all ages but particularly among older adults.^1–3^ Of all age groups, older adults are the least active, have the highest rates of inactivity-related health complications and represent the fastest-growing population worldwide.^3–5^ To reduce the individual and societal burden of non-communicable diseases (NCDs) among older adults, population-level reductions in sedentary time (ST) and increases in physical activity (PA) are needed. In an effort to inform the development of successful ST and PA interventions, researchers have sought to identify the correlates of ST and PA.

Perhaps due to an ever-growing reliance on personal motor vehicles worldwide,^6^ there has been interest in understanding the role of driving and other travel modes on PA and PA-related health outcomes. For example, in an analysis of 263 450 adults who participated in UK Biobank, adults who reported cycling to/from work compared with those who reported using cars or public transport had a 52% lower risk of cardiovascular disease (CVD) mortality and those who reported walking to/from work had a 36% lower risk of CVD mortality.^7^ These and other similar findings^8–10^ suggest that, if these associations are causal, encouraging active over passive travel may lead to increases in PA and reductions in NCD risk.

Whereas these studies have advanced our understanding of the impact that travel modes might have on activity, several limitations need to be addressed. No study has investigated whether the associations between driving and activity levels are the same in older and younger adults. Driving has been linked to lower moderate-to-vigorous-intensity PA (MVPA) and lower total PA in younger and middle-aged adults,^11^^,^^12^ but there is evidence that driving might facilitate activity during older age.^13^ This may be because having access to a car may provide the extra support older adults need to get out and about and to maintain their active lifestyles. Within-study comparisons of associations are needed to determine whether age-specific policies would be useful in facilitating activity across the lifespan. Other limitations of previous studies include a reliance on self-reported PA, cross-sectional study designs and little consideration of how driving and other modes of transport might influence all PA intensities that are relevant to health. These have constrained our understanding of how travel modes influence activity behaviours over time and our ability to set targets for intervention development and evaluation.

To strengthen the existing evidence base and to inform the development and evaluation of future interventions, particularly among older adults, we examined the prospective associations of self-reported driving with the entire range of PA intensities, including total PA, ST, light-intensity PA (LPA) and MVPA in younger (<50 years), middle-aged (50 to <65 years) and older (≥65 years) adults. Our secondary aim was to explore how activity levels in car/motor-vehicle users compare to those of walkers, cyclists, public-transport users and mixed-mode users.

## Methods

**Figure 1.**
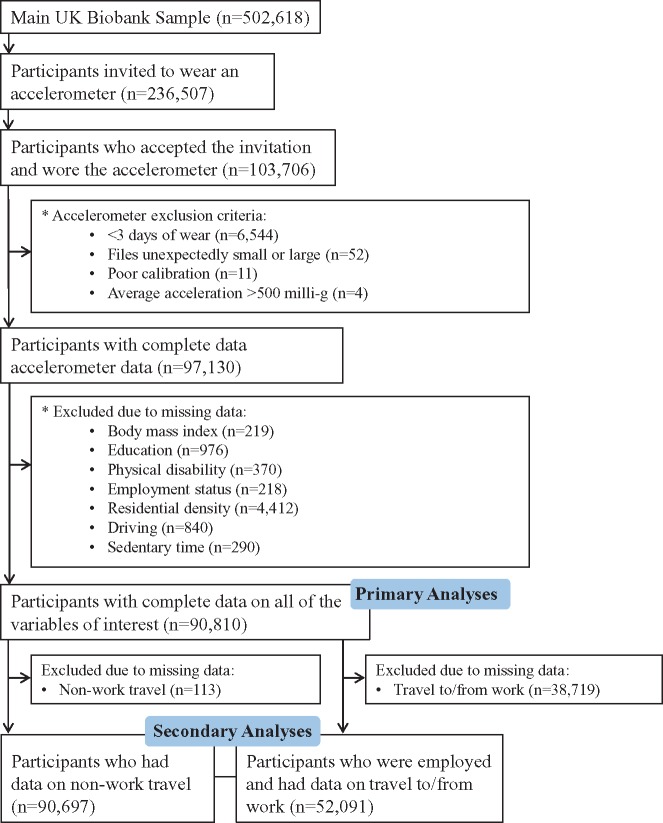
Selection of UK Biobank participants into the present study.

We used data collected as part of UK Biobank.^14^ UK Biobank is a prospective cohort of 502 618 participants who were recruited from England, Scotland and Wales and attended a baseline assessment between 2006 and 2010. The selection of participants into our study is outlined in Figure 1. In brief, participants who provided a valid e-mail address at baseline (*n* = 236** **507; 47.1%) were contacted between 2013 and 2015 and invited to wear a wrist-worn accelerometer for 7 days. Of the 236 507 participants who were invited to wear an accelerometer, 43.8% wore an accelerometer (*n* = 103 706). Of these, 87.6% (*n* = 90 810) had complete exposure and outcome data and were included in the primary analyses. Our secondary analyses were based on the sub-sets of participants who additionally had data on non-work travel (*n* = 90 697) and participants who were employed and had data on commuting modes to/from work (*n* = 52 091). With the exception of the activity data and the season variable that was derived based on the date of the accelerometer assessment, all of the variables that were included in this study were assessed at baseline. All participants provided written informed consent. UK Biobank received ethics approval from the North West Research Ethics Committee. The present study was approved by UK Biobank (Project ID: 4483).

### Driving

Participants were asked via a computer-assisted questionnaire: ‘In a typical DAY, how many hours do you spend driving?’ Responses of <0 or >24 hours/day were rejected and participants were prompted to provide another response. If the answer exceeded 6 hours/day, the participant was asked to confirm. If the participant activated the ‘Help’ button, the following message was provided: ‘If the time you spend driving varies a lot, give the average time for a 24 hour day in the last 4 weeks. Include driving a car, bus, motorcycle, boat, truck etc. Include all the driving that you do as part of work, getting to work or outside of work. If you do not drive please enter 0.’ Responses of >10 hours/day were truncated to 10 hours. We defined ‘drivers’ as participants who reported driving >0 hours/day and ‘non-drivers’ as participants who reported driving 0 hours/day.

### Travel modes

Travel modes were assessed via a computer-assisted questionnaire using the following two questions: (i) ‘In the last 4 weeks, which forms of transport have you used most often to get about? (Not including any journeys to and from work; you can select more than one answer)’ (defined in our study as ‘non-work travel’); and (ii) ‘What types of transport do you use to get to and from work? (You can select more than one answer)’ (defined in our study as ‘commuting to/from work’). For both questions, options included walking, cycling, public transport or car/motor vehicles. If the ‘Help’ button was activated for the former question, the following message was provided: ‘Remember not to include journeys to and from work.’ If the ‘Help’ button was activated for the latter question, the following message was provided: ‘If you have more than one “current job” then answer this question for your MAIN job only. If you use more than one form of transport then select all that apply.’ We coded participants who provided multiple responses as mixed-mode users and those who reported ‘none of the above’ as users of ‘other’ modes of travel.

### Accelerometer-assessed PA

#### Data collection

Wrist-worn accelerometry has been shown to be a valid method of assessing free-living activity in UK Biobank participants^15^ and in UK adults in whom walking and cycling are common.^16^ As part of this study, tri-axial accelerometers (Axivity AX3, Newcastle upon Tyne, UK) were posted to participants.^17^ Accelerometers collected data at 100 Hz with a dynamic range of ±8 g beginning at 10:00 am 2 working days after they were posted. Participants were instructed to wear their accelerometer on their dominant wrist for 7 consecutive days from the time it was received including while sleeping, bathing or swimming. After 7 days, participants returned their accelerometers to the study centre using pre-paid postage envelopes.

#### Data processing

In line with previous methods,^17–19^ the raw acceleration signals were calibrated to local gravity (1 *g*) with temperature compensation and a low-pass filter was applied to dampen machine noise. A movement-intensity signal was derived by calculating the Euclidean Norm Minus One (ENMO) metric, where one gravitational unit (1 *g*, 1 *g* = 1000 milli-*g*) is subtracted from the vector magnitude of acceleration in three axes (i.e. the Euclidean Norm), with all negative values set to 0. Non-wear time was defined as periods of ≥60 min in which the standard deviations of all three axes were <13.0 milli-*g*. We excluded participants who had <3 days of wear time, accelerometer files that were unexpectedly small or large, poorly calibrated accelerometers and/or unrealistically high average accelerations (>500 milli-*g*). Participants were required to have a minimum of 72 hours of valid wear time to ensure a stable characterization of their average daily-activity patterns.^20^ We did not apply a valid weekend day criterion, as 98.5% of participants wore their accelerometers for at least 10 hours/day on either a Saturday or a Sunday. Total PA (average ENMO) was summarized for each participant and expressed as average acceleration (mean milli-*g)*. Based on the relationship between wrist-acceleration intensity and activity energy expenditure in British adults,^15^^,^^18^ we defined ST, LPA and MVPA as wear time (5-second resolution) with ENMO values ≤30 milli-*g* (minus self-reported sleep duration), >30 and <125 milli-*g* and ≥125 milli-*g*, respectively, all expressed in min/day. Sleep duration used in the calculation of ST was assessed as part of the computer-assisted questionnaire using the following question: ‘About how many hours sleep do you get in every 24 hours? (please include naps).’ Self-reported sleep durations of <5 and >12 hours/day were assigned values of 5 and 12 hours/day, respectively.

### Covariates

Height and weight were assessed by trained research assistants following standard operating procedures and used to calculate body mass index (BMI; kg/m^2^). Age, sex, physical disability (receiving attendance allowance, disability living allowance or Blue Badge coverage), education and employment status (currently in paid employment or self-employed vs not employed/other) were assessed via a computer-assisted questionnaire completed at baseline. Residential density (permanent dwellings/km^2^) was calculated for a 500-metre street network buffer around the home address that was provided at baseline. Seasons at the baseline and accelerometer assessments were coded as two continuous periodic variables: sin(2π × day-of-year/365.25) and cos(2π × day-of-year/365.25). Follow-up time was calculated as the difference in years between the baseline and accelerometer assessments.

### Statistical analyses

Descriptive statistics were produced overall and by driving status. We used multivariable linear-regression models to estimate the prospective associations of driving and specific travel modes with total PA, ST, LPA and MVPA. We conducted analyses in the entire sample overall and stratified by age category (<50, 50 to <65, ≥65 years) and tested for interactions of the associations of interest by age in years by including the relevant interaction parameter in the adjusted models. The ST and LPA models were not log-transformed as the model assumptions were satisfied. Total PA and MVPA were transformed on the natural logarithmic scale. Mean differences in total PA (milli-*g*) and MVPA (min/day) were computed by back-transforming the regression estimates to represent the percentage differences in the outcomes. Since milli-*g* are difficult to interpret, the estimates of the total PA models were kept in units representing percentage differences in activity. The percentage differences in the MVPA were, however, divided by 100 and multiplied by the average activity in each age subgroup to represent the estimated mean difference in MVPA in minutes per day. Due to <1% of participants reporting ‘other’ as their primary mode of commuting to/from work and for non-work purposes, we did not report mean differences in activity levels for this category. We used evidence from the literature on transport and PA to inform which variables may be potential predictors of PA and/or confounders for the associations of interest.^21–27^ In our regression models, we included age, sex, BMI, education, physical disability, employment status, season at the baseline and accelerometer assessments, residential density and follow-up time after verifying that inclusion of average household income, occupational class and journey frequency in the models did not importantly alter the study findings. Whereas baseline BMI could be a mediating factor for the potential causal association between travel modes and PA, we controlled for BMI because we were interested in inferences for public health independently of BMI. To investigate how the choice of cut-points may have altered our main study findings, we conducted sensitivity analyses modelling the driving–activity associations using different intensity cut-points for LPA (i.e. 25–100 and 35–150 milli-*g*) and MVPA (i.e. ≥100 and ≥150 milli-*g*). Since one of our main exposures (i.e. non-work travel modes) may relate to journeys that are most likely to occur on weekends, we also conducted sensitivity analyses to ensure that there was no difference when including/excluding the small subset of participants (1.5%) who did not have at least 10 hours/day of valid accelerometer data on either a Saturday or a Sunday. All analyses were based on complete-case data and were conducted in Stata/SE 14.2 (College Station, TX: StataCorp LP).

## Findings

### Descriptive characteristics

Characteristics of the study population are presented overall and by driving status in Table 1. In brief, participants were on average 56.1 years old (range: 39–70) and overweight (26.7 kg/m^2^). Participants accumulated a median total PA of 27.1 milli-*g* (Interquartile range: 22.5, 32.6), an average of 641.1 min/day of ST [standard deviation (SD) = 100.3], an average of 294.6 min/day of LPA (SD = 64.2) and a median MVPA of 67.7 min/day (Interquartile range: 47.5, 95.0). Most participants were college/university-educated (70.7%) and reported at least some driving (83.1%). Overall, 62.1% of participants were employed (90.5% of those aged <50 years, 63.1% of those aged 50 to <65 years and 15.4% of those aged ≥65 years). Compared with non-drivers, drivers were more likely to be men (45.9 vs 32.9%), to have a college or university education (72.0 vs 64.4%), to not have a physical disability (2.5 vs 5.0%), to be employed (63.1 vs 57.3%) and to live in a less residentially dense neighbourhood (1903 vs 2870 permanent dwellings/km^2^).

**Table 1.** Characteristics of the study population, overall and by driving status^a^

	Overall	Non-drivers	Drivers
No. participants	90 810	15 373	75 437
Age, years	56.1 (7.8)	56.2 (7.9)	56.1 (7.8)
Body mass index, kg/m^2^	26.7 (4.5)	26.5 (4.9)	26.7 (4.4)
Women, %	56.3	67.1	54.1
Age group			
<50 years, %	23.6	23.9	23.6
50 to <65 years, %	60.7	59.8	60.9
≥65 years, %	15.7	16.3	15.5
College/university education, %	70.7	64.4	72.0
Physical disability, %	2.9	5.0	2.5
Employed , %	62.1	57.3	63.1
Residential density, permanent dwellings/km^2^	2066 (1341)	2870 (1821)	1903 (1154)
Driving time, hrs/day	0.9 (1.0)	0 (0)	1.1 (1.0)
Total PA, milli-*g*	27.1 (22.5, 32.6)	26.8 (22.0, 32.5)	27.2 (22.6, 32.6)
ST, min/day	641.1 (100.3)	649.7 (105.4)	639.4 (99.2)
LPA, min/day	294.6 (64.2)	285.2 (67.0)	296.5 (63.4)
MVPA, min/day	67.7 (47.5, 95.0)	70.6 (47.5, 97.9)	67.7 (47.5, 93.6)

a Values represent means (standard deviations) for continuous variables and percentages for categorical variables, with the exception of total PA and MVPA, which are presented as medians (interquartile ranges).

The mean follow-up time between the baseline and accelerometry assessments was 5.7 years (SD = 1.1; range: 2.8–8.6). Compared with the participants who were retained in the main analyses, those who were excluded had a higher average BMI (e.g. 27.6 vs 26.7 kg/m^2^), a greater percentage had a physical disability (e.g. 6.7 vs 2.9%), fewer were women (e.g. 54.0 vs 56.3%) and fewer had a college/university education (e.g. 57.9 vs 70.7%). Excluded participants also accumulated slightly less total PA, LPA and MVPA and were on average slightly more sedentary than included participants (e.g. 64.8 vs 67.7 min/day in MVPA; Supplementary Table 1, available as Supplementary data at *IJE* online).

The distributions of travel modes are presented in Table 2. In brief, most participants (51.2%) reported using mixed modes for non-work travel. This was followed by car/motor vehicles (36.8%), walking (6.7%), public transport (3.8%) and cycling (1.4%). For commuting to/from work, most participants reported using car/motor vehicles (60.4%), followed by mixed-mode use (23.6%), public transport (7.9%), walking (5.0%) and cycling (2.8%). Adjusted mean levels of activity in drivers and non-drivers and across travel modes are provided in Supplementary Tables 2 and 3, available as Supplementary data at *IJE* online, respectively.

**Table 2.** Self-reported travel modes by purpose, overall and by age category (%)

	Overall	<50 years	50 to <65 years	≥65 years
Non-work travel				
No. participants	90 697	21 458	55 045	14 194
Walking	6.7	6.7	6.9	5.8
Cycling	1.4	1.8	1.3	0.9
Public transport	3.8	3.2	3.7	4.7
Car/motor vehicle	36.8	38.5	37.8	30.6
Mixed mode^a^	51.2	49.6	50.2	57.9
Other	0.2	0.2	0.2	0.1
Commuting to/from work				
No. participants	52 091	18 377	32 000	1714
Walking	5.0	4.9	5.0	5.5
Cycling	2.8	3.5	2.5	2.2
Public transport	7.9	7.5	8.0	10.0
Car/motor vehicle	60.4	58.0	61.9	58.6
Mixed mode^b^	23.6	25.9	22.3	22.8
Other	0.3	0.3	0.4	0.9

a 85.8% car/motor-vehicle use + walking, cycling and/or public transport; 13.9% walking + public transport and/or cycling; 0.3% public transport + cycling.

b 76.3% car/motor-vehicle use + walking, cycling and/or public transport; 19.9% walking + public transport and/or cycling; 3.8% public transport + cycling.

### Driving

Results adjusted for different sets of covariates did not indicate marked confounding for the associations of interest (Supplementary Table 4, available as Supplementary data at *IJE* online), except for ST and LPA, in which there was some evidence of confounding by sex in older adults (sex-only adjusted models not shown). In adjusted models, drivers accumulated 1.4% more total PA (95% confidence interval (CI) 0.9, 1.9), 11.2 min/day less ST (–12.9, –9.5), 12.2 min/day more LPA (11.0, 13.3) and 0.9 min/day less MVPA (–1.6, –0.2) than non-drivers (Table 3). Patterns were similar across age groups for total PA and ST (*p* interaction = 0.252 and 0.799, respectively). There were significant interactions by age for LPA and MVPA (*p* interaction < 0.001). Among older adults, drivers accumulated 10.1 min/day more LPA than non-drivers (7.3, 12.9), whereas, among younger adults, drivers accumulated 16.3 min/day more LPA than non-drivers (13.9, 18.6).

**Table 3.** Adjusted mean differences in total PA, ST, LPA and MVPA in drivers compared with non-drivers (95% confidence intervals)^a^

	Overall	<50 years	50 to <65 years	≥65 years	*p* for interaction
No. participants	90 810	21 468	55 127	14 215	
Total PA, %	1.4 (0.9, 1.9)	1.0 (–0.1, 2.0)	1.7 (1.1, 2.3)	1.6 (0.4, 2.8)	0.252
ST, min/day	–11.2 (–12.9, –9.5)	–12.5 (–16.2, –8.8)	–10.1 (–12.4, –7.9)	–11.9 (–16.2, –7.6)	0.799
LPA, min/day	12.2 (11.0, 13.3)	16.3 (13.9, 18.6)	11.6 (10.2, 13.1)	10.1 (7.3, 12.9)	<0.001
MVPA, min/day	–0.9 (–1.5, –0.2)	–3.3 (–4.8, –1.8)	–0.4 (–1.3, 0.4)	0.2 (–1.3, 1.7)	<0.001

a Values were adjusted for age, sex, BMI, education, physical disability, employment status, season at baseline and accelerometer assessment, residential density and follow-up time.

### Non-work travel

In adjusted models, cyclists accumulated 10.7% more total PA (9.0, 12.4), 20.5 min/day less ST (–26.0, –15.0), 4.2 min/day more LPA (0.6, 7.7) and 14.5 min/day more MVPA (12.0, 17.2) than car/motor-vehicle users (Table 4). Walking was also associated with total PA, ST and MVPA (i.e. 4.2% more total PA, 7.5 min/day less ST and 10.1 min/day more MVPA). Public-transport users accumulated 2.1% less total PA (–3.0, –1.1), 15.3 min/day more ST (11.8, 18.7) and 12.0 min/day less LPA (–14.2, –9.8) than car/motor-vehicle users. Mixed-mode use was associated with 2.3% more total PA (1.9, 2.7), 3.4 min/day less ST (–4.8, –2.1) and 4.9 min/day more MVPA (4.3, 5.5). Interactions by age were observed for the mixed mode–total PA, public transport–LPA/MVPA and the walking–MVPA associations (Table 4, *p* < 0.05)—with estimated associations generally slightly smaller or in the opposite direction in older adults. For example, older public-transport users accumulated 12.4 min/day less LPA than older car/motor-vehicle users, whereas young public-transport users accumulated 17.5 min/day less LPA than younger car/motor-vehicle users; and older public-transport users accumulated 1.5 min/day less MVPA (–4.1, 1.2), whereas younger public-transport users accumulated 1.9 min/day more MVPA than car/motor-vehicle users (–1.2, 5.1).

**Table 4.** Adjusted mean differences in total PA, ST, LPA and MVPA in cyclists, walkers, mixed-mode users and public-transport users compared with car/motor-vehicle users, by travel purpose (95% confidence intervals)^a^

		Overall	<50 years	50 to <65 years	≥65 years	*p* for interaction
Non-work travel						
No. participants		90 697	21 458	55 045	14 194	
Total PA, %	Car/motor vehicle	REF	REF	REF	REF	
	Walking	4.2 (3.5, 5.0)	3.9 (2.3, 5.5)	4.1 (3.2, 5.1)	4.7 (2.6, 6.8)	0.665
	Cycling	10.7 (9.0, 12.4)	13.9 (10.8, 17.1)	9.0 (6.8, 11.2)	9.9 (4.9, 15.2)	0.053
	Public transport	–2.1 (–3.0, –1.1)	–1.9 (–4.0, 0.2)	–1.6 (–2.8, –0.4)	–3.3 (–5.4, –1.2)	0.219
	Mixed mode	2.3 (1.9, 2.7)	2.7 (1.9, 3.5)	2.2 (1.7, 2.7)	2.2 (1.2, 3.2)	0.036
ST, min/day	Car/motor vehicle	REF	REF	REF	REF	
	Walking	–7.5 (–10.2, –4.9)	–5.9 (–11.4, –0.3)	–6.9 (–10.2, –3.6)	–11.5 (–18.7, –4.3)	0.507
	Cycling	–20.5 (–26.0, –15.0)	–25.2 (–35.1, –15.3)	–20.5 (–27.7, –13.3)	–10.5 (–27.5, 6.6)	0.380
	Public transport	15.3 (11.8, 18.7)	18.5 (10.8, 26.2)	13.8 (9.4, 18.2)	16.9 (9.1, 24.8)	0.502
	Mixed mode	–3.4 (–4.8, –2.1)	–5.4 (–8.2, –2.5)	–3.1 (–4.9, –1.4)	–1.8 (–5.4, 1.8)	0.179
LPA, min/day	Car/motor vehicle	REF	REF	REF	REF	
	Walking	–4.0 (–5.7, –2.3)	–3.9 (–7.5, –0.4)	–5.0 (–7.2, –2.9)	–0.6 (–5.2, 4.1)	0.118
	Cycling	4.2 (0.6, 7.7)	0.9 (–5.5, 7.3)	5.5 (0.9, 10.1)	5.0 (–5.9, 16.0)	0.070
	Public transport	–12.0 (–14.2, –9.8)	–17.5 (–22.4, –12.5)	–10.3 (–13.2, –7.5)	–12.4 (–17.4, –7.3)	0.015
	Mixed mode	–0.6 (–1.4, 0.3)	–0.6 (–2.4, 1.2)	–0.6 (–1.7, 0.5)	–0.7 (–3.0, 1.6)	0.324
MVPA, min/day	Car/motor vehicle	REF	REF	REF	REF	
	Walking	10.1 (8.9, 11.3)	9.8 (7.3, 12.2)	9.7 (8.3, 11.2)	9.6 (6.7, 12.6)	0.013
	Cycling	14.5 (12.0, 17.2)	19.5 (14.8, 24.6)	12.9 (9.7, 16.3)	12.2 (5.3, 19.8)	0.623
	Public transport	0.7 (–0.7, 2.1)	1.9 (–1.2, 5.1)	1.5 (–0.1, 3.3)	–1.5 (–4.1, 1.2)	0.006
	Mixed mode	4.9 (4.3, 5.5)	5.5 (4.3, 6.8)	5.0 (4.3, 5.7)	4.3 (2.9, 5.6)	0.813
Commuting to/from work						
No. participants		52 091	18 377	32 000	1714	
Total PA, %	Car/motor vehicle	REF	REF	REF	REF	
	Walking	3.4 (2.3, 4.5)	4.4 (2.5, 6.3)	3.0 (1.6, 4.4)	–2.2 (–7.6, 3.6)	0.108
	Cycling	9.7 (8.2, 11.2)	8.9 (6.6, 11.3)	10.4 (8.4, 12.5)	6.1 (–2.7, 15.8)	0.637
	Public transport	–1.5 (–2.3, –0.6)	–2.3 (–3.8, –0.8)	–1.0 (–2.1, 0.1)	–1.9 (–6.2, 2.6)	0.136
	Mixed mode	2.0 (1.5, 2.6)	1.6 (0.7, 2.6)	2.4 (1.6, 3.1)	–0.3 (–3.4, 3.0)	0.738
ST, min/day	Car/motor vehicle	REF	REF	REF	REF	
	Walking	–7.4 (–11.2, –3.6)	–7.0 (–13.6, –0.4)	–9.0 (–13.8, –4.1)	15.0 (–5.3, 35.3)	0.212
	Cycling	–17.0 (–22.0, –12.1)	–8.8 (–16.5, –1.1)	–23.4 (–30.1, –16.8)	–17.3 (–47.9, 13.4)	0.014
	Public transport	15.6 (12.5, 18.8)	24.6 (19.0, 30.1)	10.9 (6.9, 14.9)	14.7 (–1.0, 30.5)	<0.001
	Mixed mode	1.8 (–0.8, 3.8)	6.0 (2.7, 9.4)	–0.7 (–3.3, 1.9)	1.9 (–9.4, 13.2)	<0.001
LPA, min/day	Car/motor vehicle	REF	REF	REF	REF	
	Walking	–6.1 (–8.5, –3.6)	–6.3 (–10.6, –2.1)	–5.3 (–8.4, –2.1)	–18.5 (–31.8, –5.1)	0.949
	Cycling	0.8 (–2.4, 4.0)	–8.7 (–13.7, –3.8)	7.7 (3.3, 12.0)	3.7 (–16.5, 24.0)	<0.001
	Public transport	–14.0 (–16.1, –12.0)	–20.5 (–24.0, –16.9)	–10.8 (–13.3, –8.2)	–11.2 (–21.6, –0.8)	<0.001
	Mixed mode	–5.9 (–7.2, –4.6)	–10.4 (–12.5, –8.2)	–3.3 (–5.0, –1.7)	–4.5 (–12.0, 2.9)	<0.001
MVPA, min/day	Car/motor vehicle	REF	REF	REF	REF	
	Walking	8.9 (7.2, 10.7)	10.5 (7.6, 13.6)	8.4 (6.3, 10.5)	1.8 (–5.2, 9.7)	0.499
	Cycling	14.1 (11.8, 16.5)	14.0 (10.5, 17.7)	15.1 (12.1, 18.4)	4.0 (–6.6, 16.9)	0.216
	Public transport	2.5 (1.2, 3.8)	2.6 (0.4, 4.9)	2.6 (1.1, 4.3)	0.2 (–5.2, 6.1)	0.458
	Mixed mode	5.6 (4.7, 6.5)	5.6 (4.2, 7.0)	5.8 (4.7, 6.9)	2.3 (–1.8, 6.6)	0.501

a Values represent mean differences (95% confidence interval) in the PA variables according to each specific travel mode in comparison to car/motor-vehicle use, adjusted for age, sex, BMI, education, physical disability, employment status, season at baseline and accelerometer assessment, residential density and follow-up time.

### Commuting to/from work

In adjusted models, cyclists accumulated 9.7% more total PA (8.2, 11.2), 17.0 min/day less ST (–22.0, –12.1) and 14.1 min/day more MVPA (11.8, 16.5) than car/motor-vehicle users (Table 4). Walking was associated with 3.4% more total PA (2.3, 4.5), 7.4 min/day less ST (–11.2, –3.6) and 8.9 min/day more MVPA (7.2, 10.7). Public-transport users accumulated 1.5% less total PA (–2.3, –0.6), 15.6 min/day more ST (12.5, 18.8) and 14.0 min/day less LPA (–16.1, –12.0). Mixed-mode use was associated with 2.0% more total PA (1.5, 2.6) and 5.6 min/day more MVPA (4.7, 6.5). Interactions by age were observed for the cycling/public transport/mixed mode use–ST/LPA associations, although these interactions are likely a consequence of only 15.4% of participants being employed after the age of 65 years (Table 4, *p* < 0.05).

### Sensitivity analyses

Our main study findings were robust to the application of different intensity cut-points, although higher cut-points attenuated the observed associations for ST and LPA (Supplementary Table 5, available as Supplementary data at *IJE* online). None of our results were importantly changed by the exclusion of the 1.5% of participants who did not have at least 10 hours/day of valid accelerometer data on either a Saturday or a Sunday (data not shown).

## Discussion

In our study, we found that drivers accumulated 1.4% more total PA, 11.2 min/day less ST, 12.2 min/day more LPA and 0.9 min/day less MVPA than non-drivers. We also demonstrated differences in activity levels across modes of travel for non-work purposes and for commuting to/from work. For example, for non-work travel, cyclists accumulated approximately 10.7% more total PA, 20.5 min/day less ST and 14.5 min/day more MVPA than car/motor-vehicle users; and walkers accumulated approximately 4.2% more total PA, 7.5 min/day less ST and 10.1 min/day more MVPA compared with car/motor-vehicle users. We also observed some beneficial associations for mixed-mode users (e.g. mixed-mode users accumulated 2.3% more total PA). Public-transport use appeared to have detrimental associations with activity compared with car/motor-vehicle use (e.g. public-transport use for non-work travel: 15.3 min/day more ST). Similar differences in activity were observed for commuting to/from work. Whereas we observed some interactions by age for the commuting to/from work models, these were likely due to the inconclusive associations that resulted from the small percentage of older adults who were still employed after the age of 65. The other interactions that we observed by age were small, with estimated differences generally consistent across age groups.

Cross-sectional studies on the associations between driving and activity in younger and middle-aged adults have found that car access/use is associated with lower levels of activity.^12^^,^^28^ For example, in an analysis of data from 2101 adults aged 25–45 years, US and non-US (from the Seychelles, Jamaica, South Africa and Ghana) car owners accumulated 24.3 and 9.7 min/day less accelerometer-assessed MVPA than non-car owners, respectively (20.7 vs 45.1 min/day; 24.9 vs 34.6 min/day).^12^ In our analyses of younger adults (i.e. <50 years), we did not find any conclusive difference in total PA between drivers and non-drivers, but we did find that drivers accumulated 3.3 min/day less MVPA than non-drivers. The smaller differences in MVPA observed in our study compared with the US analyses may be a result of our study population being older (i.e. we did not include adults <39 years) or UK car owners relying more heavily on public transport and/or active travel than US adults^29^^,^^30^—both of which may attenuate driving–MVPA associations. In contrast to negative driving–activity associations that have been reported in studies of younger and middle-aged adults, null or positive driving–PA associations have been reported in studies of older adults.^13^^,^^31–33^ For example, in an analysis of 880 older US adults (mean age = 75.0 years), no difference was observed in accelerometer-assessed MVPA between drivers and non-drivers (10.9 vs 11.0 min/day, respectively; *p* > 0.05)^32^ and, in an analysis of 214 older UK adults (mean age = 78.1 years), each weekly car trip as a driver was associated with 166 more steps/day.^13^ In line with the US study, we did not find any conclusive differences between older drivers and non-drivers in MVPA [0.2 min/day, 95% CI −1.3, 1.7] and, in line with the UK study, we found that older drivers accumulated 1.6% more total PA than older non-drivers (95% CI 0.4, 2.8).

Activity levels in users of different modes of travel have been examined previously^34–37^ but our study is the first, to our knowledge, to use objective activity monitoring to determine whether age-specific associations exist across the entire range of activity intensities and to determine whether these associations apply to travel modes for work and non-work purposes. We identified clinically important differences in activity levels across modes of travel for non-work purposes and for commuting to/from work. For example, for non-work travel, cyclists accumulated approximately 15 min/day more MVPA than car/motor-vehicle users. Similar differences in MVPA have been associated with a lower risk of death due to any causes^38–40^ [e.g. each additional 10 min of accelerometer-assessed MVPA has been linked to a 8% decreased risk of mortality among older men (hazard ratio 0.92, 95% CI 0.86, 0.98)].^38^ When interpreted in light of the driving–activity associations, our findings suggest that assessing specific travel modes rather than driving status alone may better capture variations in activity and thus also provides more useful information for intervention development.

Whereas we found cycling, walking and, to some extent, mixed-mode use had beneficial associations with activity, we found that (for non-work travel) users of public transport accumulated approximately 2% less total PA, 12 min/day less LPA and 15 min/day more ST than car/motor-vehicle users. The estimated effects were similar for commuting to/from work. Although there are exceptions,^41^ public-transport users have generally been demonstrated to accumulate higher levels of PA^34–36^^,^^42^^,^^43^ and/or to be more likely to meet the recommended levels of daily activity^36^^,^^44^^,^^45^ than car users. This is thought to be due to public-transport users walking or using other modes of active travel to access public transit.^41^^,^^44^^,^^46^ The divergent findings between our study and those of previous studies may be attributable to differences in activity measurement or population-specific differences in the activity levels of car and public-transport users. For example, car users may be more active in our study population than in the previous study populations, thereby attenuating observable differences between car and public-transport users. To further elucidate the role of public transport on PA, more research using objective measures of PA in different populations and across different activity intensities is encouraged.

Strengths of our study include the objective assessment of activity across the entire intensity spectrum, the large sample size that enabled comparisons of association across age groups and the availability of data on both driving and other travel modes. An additional strength is the prospective study design. Given that present activity predicts future activity,^47^^,^^48^ the non-contemporaneous assessment of exposure and outcome demonstrates either how well travel modes and activity behaviours track over time and/or the impact that present travel modes have on future activity behaviours. Three limitations should also be noted. Previous work has suggested that UK Biobank participants may be healthier than the general British population.^49^ The UK Biobank participants included in our analysis were more affluent and more active than the UK Biobank participants who were excluded. As a result, the degree to which we can generalize our findings to the general UK population and to the full UK Biobank sample is limited. Second, the outcomes in this study represent cumulative activity and thus cannot be interpreted in the context of activity guidelines that are based on activity accumulated in bouts of at least 10 minutes. We chose to use cumulative activity given the variation in how activity has been defined in previous work^50^ and because cumulative activity has been shown to be important for health.^51^^,^^52^ Third, we only examined effect modification by age. There may be other effect modifiers of the relationships of interest (e.g. urban/rural residence). Researchers are encouraged to consider the role of these factors in future analyses.

In conclusion, this is the first prospective study to quantify differences in accelerometer-assessed total PA, ST, LPA and MVPA across different travel modes in young, middle-aged and older adults. Our study demonstrates that walking, cycling and, to a lesser degree, mixed-mode use are associated with more optimal activity profiles in adults of all ages. Our study also provides targets for intervention development and evaluation by estimating differences in activity that can be expected across different travel modes. For example, if causal, it may be possible to decrease ST by approximately 20 min/day and to increase MVPA by approximately 15 min/day by encouraging adults who normally use cars/motor vehicles for non-work travel to use bicycles instead. Lastly, our study demonstrates that assessing specific travel modes rather than driving status may better capture variations in activity and that, whereas there may be some age-specific differences in the associations between travel modes and activity, these differences appear small.

## Funding

This work was supported by the Lifelong Health and Wellbeing Cross-Council Programme (MR/K025147/1 to S.J.G.), the Medical Research Council (MRC) (MC_UU_12015/4 to SJG, MC_UP_12015/6 to D.O. and J.P., and MC_UU_12015/3 to S.B. and K.W.), the Canadian Institutes of Health Research (FRN 146766; Fellowship to S.H.), and MedImmune (Studentship to T.W.). The work was supported by the Centre for Diet and Activity Research (CEDAR)—a UKCRC Public Health Research Centre of Excellence that is funded by the British Heart Foundation, Cancer Research UK, Economic and Social Research Council, Medical Research Council, the National Institute for Health Research and the Wellcome Trust. The University of Cambridge has received salary support in respect of S.J.G. from the NHS in the East of England through the Clinical Academic Reserve. The views expressed are those of the authors and not necessarily those of the NHS or the Department of Health. This research has been conducted using the UK Biobank Resource under Application Number 4483.

## Supplementary Material

dyz065_Supplementary_Data
