# Supplementary material for: Driving status, travel modes and accelerometer-assessed physical activity in younger, middle-aged and older adults: a prospective study of 90 810 UK Biobank participants
Source: Int J Epidemiol. 2019 Apr 19;48(4):1175–86. doi: 10.1093/ije/dyz065 (PMC6693808; doi:10.1093/ije/dyz065)
Supplement: dyz065_Supplementary_Data [file dyz065_supplementary_data.docx]

| **Supplementary Table 1.** Basic characteristics of the UK Biobank participants who were excluded from the main analyses due to missing exposure/outcome/covariate data (Group A), the UK Biobank participants who wore the accelerometers but were excluded from the main analyses due to missing exposure/outcome/covariate data (Group B^a^), and the UK Biobank participants who were included in the main analysis (Final Sample).^b^ | | | |
| --- | --- | --- | --- |
|  | **Group A^c^** | **Group B^d,e^** | **Final Sample** |
| No. Participants | 411,808 | 6,320 | 90,810 |
|  |  |  |  |
| Age, years | 56.6 (8.2) | 56.3 (7.8) | 56.1 (7.8) |
| Body mass index, kg/m^2^ | 27.6 (4.8) | 26.7 (4.7) | 26.7 (4.5) |
| Women, % | 54.0 | 55.7 | 56.3 |
| College/university education, % | 57.9 | 69.9 | 70.7 |
| Physical disability, % | 6.7 | 5.1 | 2.9 |
| Employed , % | 56.5 | 58.9 | 62.1 |
| Residential density, residences/km^2^ | 2,148 (1,298) | 2,247 (1,397) | 2,066 (1,341) |
| Driving time, hrs/day | 1.0 (1.2) | 0.8 (1.0) | 0.9 (1.0) |
|  |  |  |  |
| Total PA, milli-g | - | 26.2 (21.6, 31.9) | 27.1 (22.5, 32.6) |
| ST, min/day | - | 650.4 (104.3) | 641.1 (100.3) |
| LPA, min/day | - | 289.3 (67.1) | 294.6 (64.2) |
| MVPA, min/day | - | 64.8 (43.2, 92.2) | 67.7 (47.5, 95.0) |

^a^ Excluded participants with <3 days of wear time, accelerometry files that were unexpectedly small or large, poorly calibrated accelerometers, and/or average accelerometer >500 milli-gravities (milli-g).

^b^ Values represent means (standard deviations) for continuous variables and percentages for categorical variables with the exception of total PA and MVPA which are presented as medians (interquartile ranges).

^c^ Sample size of Group A: N=502,618–90,810=411,808; BMI (n=408,703); college/university education (n=401,666); physical disability (n=406,272); employed (n=408,850); residential density (n=390,291); driving time (n= 401,870)

^d^ Sample size of Group B: N=97,130–90,810=6,320; BMI (n=6,101); college/university education (n=5,344); physical disability (n=5,950); employed (n=6,102); residential density (n=1,908); driving time (n=5,480); ST (n=6,030)

^e^ Abbreviations: PA, physical activity; ST, sedentary time; LPA, low-intensity physical activity; MVPA, moderate-to-vigorous intensity physical activity.

| **Supplementary Table 2. Adjusted mean levels of total PA, ST, LPA, and MVPA in drivers and non-drivers (95% confidence intervals).** | | | | |
| --- | --- | --- | --- | --- |
|  | **Overall** | **<50 years** | **≥50<65 years** | **≥65 years** |
| No. Participants | 90,810 | 21,468 | 55,127 | 14,215 |
| Total PA, milli-g |  |  |  |  |
| Non-drivers | 26.6 (26.5, 26.8) | 29.7 (29.4, 30.0) | 26.4 (26.2, 26.5) | 23.4 (23.1, 23.6) |
| Drivers | 27.0 (27.0, 27.1) | 30.0 (29.9, 30.1) | 26.8 (26.8, 26.9) | 23.8 (23.6, 23.9) |
| ST, min/day |  |  |  |  |
| Non-drivers | 650.4 (648.9, 652.0) | 636.5 (633.2, 639.8) | 651.9 (649.8, 653.9) | 664.7 (660.8, 668.5) |
| Drivers | 639.2 (638.5, 639.9) | 624.0 (622.5, 625.4) | 641.7 (640.9, 642.6) | 652.7 (651.0, 654.5) |
| LPA, min/day |  |  |  |  |
| Non-drivers | 284.5 (283.5, 285.5) | 283.0 (280.9, 285.1) | 285.5 (284.2, 286.8) | 281.3 (278.8, 283.8) |
| Drivers | 296.7 (296.2, 297.1) | 299.3 (298.4, 300.2) | 297.1 (296.6, 297.7) | 291.4 (290.2, 292.5) |
| MVPA, min/day |  |  |  |  |
| Non-drivers | 64.8 (64.2, 65.3) | 82.3 (81.1, 83.6) | 64.0 (63.3, 64.7) | 47.3 (46.2, 48.5) |
| Drivers | 64.0 (63.8, 64.2) | 79.2 (78.7, 79.8) | 63.6 (63.3, 63.9) | 47.5 (47.0, 48.0) |

^a^ Abbreviations: PA, physical activity; ST, sedentary time; LPA, low-intensity physical activity; MVPA, moderate-to-vigorous intensity physical activity; Adjusted for age, sex, BMI, education, physical disability, employment status, season at baseline and accelerometer assessment, residential density, and follow-up time.

| **Supplementary Table 3. Adjusted means levels of total PA, ST, LPA, and MVPA in participants, by travel purpose (95% confidence intervals).** | | | | | |
| --- | --- | --- | --- | --- | --- |
|  |  | **Overall** | **<50 years** | **≥50<65 years** | **≥65 years** |
| **Non-work travel** | | | | | |
| No. Participants |  | 90,697 | 21,458 | 55,045 | 14,194 |
| Total PA, milli-g | Car/motor vehicles | 26.7 (25.6, 28.0) | 30.9 (28.2, 33.9) | 26.1 (24.7, 27.6) | 23.1 (20.1, 26.7) |
|  | Walking | 27.7 (27.5, 27.9) | 30.6 (30.1, 31.0) | 27.5 (27.3, 27.7) | 24.5 (24.0, 24.9) |
|  | Cycling | 29.4 (29.0, 29.9) | 33.5 (32.6, 34.4) | 28.8 (28.2, 29.3) | 25.7 (24.5, 26.9) |
|  | Public transport | 26.0 (25.8, 26.3) | 28.9 (28.3, 29.5) | 26.0 (25.7, 26.3) | 22.6 (22.2, 23.1) |
|  | Mixed mode | 27.2 (27.1, 27.3) | 30.2 (30.1, 30.4) | 27.0 (26.9, 27.1) | 23.9 (23.8, 24.0) |
|  |  |  |  |  |  |
| ST, min/day | Car/motor vehicles | 634.8 (618.6, 651.1) | 617.1 (584.0, 650.2) | 634.9 (614.8, 654.9) | 675.0 (623.3, 726.7) |
|  | Walking | 635.5 (633.1, 637.9) | 623.1 (618.1, 628.2) | 638.3 (635.2, 641.3) | 644.2 (637.6, 650.7) |
|  | Cycling | 622.5 (617.1, 627.9) | 603.8 (594.2, 613.5) | 624.7 (617.6, 631.7) | 645.1 (628.4, 661.9) |
|  | Public transport | 658.3 (655.0, 661.6) | 647.5 (640.2, 654.8) | 658.9 (654.7, 663.1) | 672.6 (665.3, 679.8) |
|  | Mixed mode | 639.6 (638.7, 640.4) | 623.7 (621.8, 625.5) | 642.0 (640.9, 643.2) | 653.8 (651.8, 655.9) |
|  |  |  |  |  |  |
| LPA, min/day | Car/motor vehicles | 299.5 (289.1, 310.0) | 317.4 (296.2, 338.7) | 294.6 (281.8, 307.5) | 285.3 (252.1, 318.4) |
|  | Walking | 291.6 (290.1, 293.2) | 293.7 (290.4, 296.9) | 291.2 (289.2, 293.1) | 290.2 (286.0, 294.4) |
|  | Cycling | 299.8 (296.3, 303.2) | 298.5 (292.3, 304.7) | 301.7 (297.2, 306.2) | 295.7 (285.0, 306.5) |
|  | Public transport | 283.7 (281.6, 285.7) | 280.1 (275.4, 284.8) | 285.9 (283.2, 288.6) | 278.4 (273.7, 283.0) |
|  | Mixed mode | 295.1 (294.5, 295.6) | 297.0 (295.8, 298.2) | 295.6 (294.9, 296.3) | 290.0 (288.7, 291.4) |
|  |  |  |  |  |  |
| MVPA, min/day | Car/motor vehicles | 60.4 (55.4, 65.9) | 78.8 (67.7, 91.7) | 59.4 (53.4, 66.0) | 40.7 (29.5, 56.0) |
|  | Walking | 69.8 (69.0, 70.8) | 85.0 (83.1, 87.0) | 69.1 (68.0, 70.2) | 52.8 (50.7, 55.0) |
|  | Cycling | 73.5 (71.5, 75.7) | 93.6 (89.5, 97.8) | 71.8 (69.1, 74.5) | 54.9 (49.5, 60.9) |
|  | Public transport | 62.0 (60.9, 63.1) | 78.2 (75.6, 80.9) | 62.2 (60.9, 63.6) | 43.9 (42.0, 46.0) |
|  | Mixed mode | 65.5 (65.2, 65.8) | 81.4 (80.7, 82.1) | 65.1 (64.7, 65.5) | 48.6 (47.9, 49.2) |
| **Commuting to/from work** | | | | | |
| No. Participants |  | 52,091 | 18,377 | 32,000 | 1,714 |
| Total PA, milli-g | Car/motor vehicles | 27.1 (26.0, 28.2) | 29.8 (27.6, 32.1) | 26.3 (25.1, 27.6) | 21.8 (19.0, 24.9) |
|  | Walking | 28.9 (28.6, 29.2) | 31.0 (30.5, 31.6) | 28.0 (27.6, 28.4) | 23.6 (22.3, 24.9) |
|  | Cycling | 30.6 (30.2, 31.1) | 32.4 (31.7, 33.0) | 30.0 (29.5, 30.6) | 25.6 (23.5, 27.8) |
|  | Public transport | 27.5 (27.3, 27.7) | 29.0 (28.6, 29.4) | 26.9 (26.6, 27.2) | 23.6 (22.7, 24.6) |
|  | Mixed mode | 28.5 (28.4, 28.6) | 30.2 (30.0, 30.4) | 27.8 (27.6, 28.0) | 24.0 (23.4, 24.7) |
|  |  |  |  |  |  |
| ST, min/day | Car/motor vehicles | 644.4 (630.6, 658.3) | 628.1 (600.8, 655.3) | 649.6 (632.5, 666.7) | 673.8 (626.3, 721.3) |
|  | Walking | 633.2 (629.5, 636.8) | 619.9 (613.6, 626.2) | 638.4 (633.7, 643.0) | 674.4 (655.2, 693.6) |
|  | Cycling | 623.5 (618.7, 628.4) | 618.1 (610.7, 625.6) | 623.9 (617.3, 630.4) | 642.1 (612.1, 672.1) |
|  | Public transport | 656.2 (653.3, 659.2) | 651.5(646.4, 656.6) | 658.2 (654.5, 661.9) | 674.1 (659.8, 688.4) |
|  | Mixed mode | 642.4 (640.7, 644.1) | 633.0 (630.3, 635.7) | 646.6 (644.4, 648.8) | 661.3 (651.9, 670.7) |
|  |  |  |  |  |  |
| LPA, min/day | Car/motor vehicles | 291.2 (282.2, 300.2) | 297.6 (280.0, 315.1) | 291.7 (280.6, 302.8) | 274.0 (242.6, 305.4) |
|  | Walking | 293.0 (290.6, 295.4) | 294.2 (290.1, 298.3) | 293.2 (290.2, 296.2) | 278.1 (265.5, 290.8) |
|  | Cycling | 299.9 (296.7, 303.0) | 291.8 (287.0, 296.6) | 306.1 (301.9, 310.4) | 300.4 (280.5, 320.2) |
|  | Public transport | 285.0 (283.1, 286.9) | 280.1 (276.8, 283.4) | 287.7 (285.3, 290.1) | 285.4 (276.0, 294.9) |
|  | Mixed mode | 293.2 (292.1, 294.3) | 290.1 (288.4, 291.9) | 295.1 (293.7, 296.5) | 292.1 (285.9, 298.3) |
|  |  |  |  |  |  |
| MVPA, min/day | Car/motor vehicles | 63.8 (59.5, 68.5) | 79.4 (70.1, 89.9) | 60.1 (55.0, 65.7) | 38.5 (28.6, 51.9) |
|  | Walking | 76.1 (74.7, 77.5) | 86.8 (84.3, 89.3) | 72.1 (70.4, 73.9) | 48.9 (43.4, 55.2) |
|  | Cycling | 80.6 (78.6, 82.6) | 89.9 (86.9, 93.0) | 78.0 (75.3, 80.7) | 50.9 (42.1, 61.4) |
|  | Public transport | 70.5 (69.5, 71.6) | 79.8 (78.0, 81.7) | 67.2(65.9, 68.5) | 47.6 (43.5, 52.1) |
|  | Mixed mode | 73.2 (72.6, 73.9) | 82.4 (81.4, 83.4) | 69.9 (69.1, 70.7) | 49.4 (46.6, 52.4) |

^a^ Abbreviations: PA, physical activity; ST, sedentary time; LPA, low-intensity physical activity; MVPA, moderate-to-vigorous intensity physical activity. Values represent means (95% confidence intervals); Adjusted for age, sex, BMI, education, physical disability, employment status (only in the non-work travel models), season at baseline and accelerometer assessment, residential density, and follow-up time.

| **Supplementary Table 4. Unadjusted and partially adjusted mean differences (95% confidence intervals) in total PA, ST, LPA, and MVPA in drivers compared to non-drivers.**^a,b^ | | | | | |
| --- | --- | --- | --- | --- | --- |
|  |  | **Overall** | **<50 years** | **≥50<65 years** | **≥65 years** |
| No. Participants |  | 90,810 | 21,468 | 55,127 | 14,215 |
| Total PA, % | Unadjusted | 1.9 (1.4, 2.4) | 1.2 (0.2, 2.2) | 2.3 (1.7, 3.0) | 1.0 (-0.2, 2.2) |
|  | Adjusted | 2.2 (1.7, 2.7) | 1.3 (0.3, 2.4) | 2.7 (2.0, 3.3) | 2.2 (0.9, 3.5) |
| ST, min/day | Unadjusted | -10.3 (-12.0, -8.6) | -12.8 (-16.4, -9.3) | -9.8 (-12.1, -7.6) | -8.0 (-12.3, -3.8) |
|  | Adjusted | -14.2 (-15.9, -12.5) | -14.7 (-18.2, -11.2) | -13.7 (-15.9, -11.5) | -14.5 (-18.9, -10.2) |
| LPA, min/day | Unadjusted | 11.4 (10.3, 12.5) | 17.5 (15.2, 19.8) | 11.0 (9.6, 12.5) | 3.2 (0.4, 6.1) |
|  | Adjusted | 11.4 (10.3, 12.5) | 18.4 (16.2, 20.7) | 14.6 (13.2, 16.0) | 11.8 (9.0, 14.6) |
| MVPA, min/day | Unadjusted | -0.3 (-1.0, 0.4) | -3.8 (-5.2, -2.4) | 0.4 (-0.4, 1.3) | 0.7 (-0.8, 2.3) |
|  | Adjusted | -0.3 (-1.0, 0.4) | -3.6 (-5.0, -2.2) | 0.3 (-0.6, 1.2) | 0.7 (-0.9, 2.3) |

^a^ Abbreviations: PA, physical activity; ST, sedentary time; LPA, low-intensity physical activity; MVPA, moderate-to-vigorous intensity physical activity.

^b^ Adjusted for age and sex.

| **Supplementary Table 5. Adjusted mean differences in ST, LPA, and MVPA between drivers and non-drivers using different activity cut-points (95% confidence intervals).** ^a,b^ | | | | | |
| --- | --- | --- | --- | --- | --- |
|  | **Overall** | **<50 years** | **≥50<65 years** | **≥65 years** | **p for interaction** |
| No. Participants | 90,810 | 21,468 | 55,127 | 14,215 |  |
| ST, min/day |  |  |  |  |  |
| <25 milli-g | -13.6 (-15.4, -11.8) | -15.6 (-19.4,-11.9) | -12.5 (-14.8, -10.2) | -13.6 (-18.0, -9.2) | 0.559 |
| <35 milli-g | -9.0 (-10.7, -7.3) | -9.5 (-13.1, -6.0) | -8.0 (-10.2, -5.8) | -10.4 (-14.7, -6.2) | 0.265 |
| LPA, min/day |  |  |  |  |  |
| ≥25<100 milli-g | 13.7 (12.7, 14.8) | 18.4 (16.2, 20.6) | 13.2 (11.8, 14.5) | 11.1 (8.4, 13.7) | <0.001 |
| ≥35<150 milli-g | 9.9 (8.8, 11.1) | 13.4 (10.9, 15.8) | 9.5 (8.0, 10.9) | 8.5 (5.7, 11.4) | 0.001 |
| MVPA, min/day |  |  |  |  |  |
| ≥100 milli-g | -0.01 (-0.9, 0.9) | -2.3 (-4.1, -0.4) | 0.5 (-0.5, 1.7) | 0.8 (-1.2, 2.9) | <0.001 |
| ≥150 milli-g | -0.8 (-1.4, -0.3) | -3.2 (-4.3, -2.0) | -0.4 (-1.1, 0.2) | 0.3 (-0.8, 1.4) | <0.001 |

^a^ Abbreviations: ST, sedentary time; LPA, low-intensity physical activity; MVPA, moderate-to-vigorous intensity physical activity. Statistics were adjusted for age, sex, BMI, education, physical disability, employment status, season at baseline and accelerometer assessment, residential density, and follow-up time.
